# Why is Southern African canine babesiosis so virulent? An evolutionary perspective

**DOI:** 10.1186/1756-3305-4-51

**Published:** 2011-04-13

**Authors:** Barend L Penzhorn

**Affiliations:** 1 Department of Veterinary Tropical Diseases, Faculty of Veterinary Science, University of Pretoria, Private Bag X04, Onderstepoort, 0110, Republic of South Africa

## Abstract

Canine babesiosis is a common, highly virulent disease in Southern Africa with even pups and juveniles being severely affected. This contrasts with bovine babesiosis, for example, where host, parasite and vector co-evolved and young animals develop immunity after infection without showing clinical signs. *Babesia rossi*, the main causative organism of canine babesiosis in sub-Saharan Africa, was first described from a side-striped jackal (*Canis adustus*) in Kenya. Although data are meagre, there is evidence that indigenous African canids, such as jackals and wild dogs (*Lycaon pictus*), can harbour the parasite without showing untoward effects. Dogs are not indigenous to Africa. The vast majority of dogs presented at veterinary facilities in South Africa represent recently introduced European, Asian or American breeds. The contention is that *B. rossi *is a new challenge to which these dogs have not adapted. With intensive treatment of clinical cases, natural selection is effectively negated and the status quo will probably be maintained indefinitely. It is postulated that *Babesia vogeli*, which frequently results in unapparent infections or mild manifestations in dogs, represents or is closely related to the ancestral form of the canine parasite, possibly originating from wolves (*Canis lupus*).

## Introduction

Babesiosis is one of the most important canine diseases in South Africa. Countrywide, babesiosis is diagnosed in around 10% of dogs presented to veterinary practices [1]. At the Onderstepoort Veterinary Academic Hospital on the outskirts of Pretoria, South Africa, around 12% of sick dogs presented are diagnosed with babesiosis, and around 31% of these are admitted for more intensive treatment [2]. Canine babesiosis, referred to as "malignant jaundice or bilious fever", was first reported from the Cape Colony in 1893 [3]. Early reports alluded to the virulent nature of the disease in South Africa [4-6], which differed from the manifestation in other parts of the world. Working with a South African isolate, Nutall and Hadwen (1909) commented that the parasites used in experiments in Italy must have been much less virulent than theirs [7,8]. Furthermore, dogs that survived infection with a French isolate were fully susceptible to the South African one, prompting Laveran & Nattan-Larrier to conclude in 1913 that the African babesia of dogs, if not a separate species, was at least a variety distinct from the French one [9,10].

It was soon evident that isolates from different geographic regions were vector-specific. South African isolates were transmitted by *Haemaphysalis elliptica*, previously misidentified as *Haemaphysalis leachi *[4,11]. Isolates from North Africa, the Middle East and India were transmitted by *Rhipicephalus sanguineus*, while those from Southern Europe were transmitted by *Dermacentor reticulatus *[12-14]. Later research confirmed this vector-specificity [15,16].

It is rather curious, therefore, that generally no notice was taken of this clear evidence of distinct biological differences between region-specific isolates. Until 20-odd years ago, it was generally accepted that babesiosis in dogs was caused by two species: a large piroplasm, *Babesia canis*, and a small one, *Babesia gibsoni*. Although the parasites had some characteristics in common, the disease manifestations were distinct. In 1989, Uilenberg *et al. *[17] reminded the scientific community that three distinct taxa were generally lumped under the "large" *Babesia*: *B. canis *(*sensu stricto*) [18], transmitted by *Dermacentor reticulatus*, occurring in Southern Europe, but seemingly spreading northwards [19,20]; the cosmopolitan *B. vogeli *[21], transmitted by *Rhipicephalus sanguineus*; and *B. rossi *[22], transmitted by *Haemaphysalis elliptica*, restricted to sub-Saharan Africa. It was subsequently demonstrated that these three taxa can de differentiated molecularly as well [23]. A fourth, as yet unnamed, large *Babesia *has since been reported from dogs in the USA [24].

Pre-1990 literature on canine babesiosis should therefore be considered cautiously, as the actual causative agent may be in doubt. Although *B. vogeli *has been recorded in South Africa, it seems to be relatively rare and the large body of literature on canine babesiosis, especially concerning pathogenesis and clinical manifestation, can be safely attributed to *B. rossi *[25,26]. Similarly, most references from Europe probably refer to *B. canis *(*sensu stricto*). Elsewhere, *B. vogeli *would probably be the causative agent. Persisting in merely referring to "canine babesiosis" without specifying the causative agent, is scientifically unsound.

The "small" piroplasm does not refer to a single species either. At least five taxa have been described: *Babesia gibsoni *(*sensu stricto*) [27], *Babesia conradae *[28], a *Babesia microti*-like organism [29], *Theileria annae *[30] and an unnamed *Theileria *species [31].

### Clinical manifestation of *B. rossi *infection

The earliest scientific descriptions of canine babesiosis in South Africa are of a highly virulent disease [4-6]. Subsequent intensive study has borne this out.

*Babesia rossi *causes peracute and acute disease, the clinical signs including pale mucous membranes, depression, tachycardia, tachypnoea, anorexia, weakness, splenomegaly and fever [32]. The clinical signs are attributed to tissue hypoxia resulting from anaemia and a concomitant systemic inflammatory response syndrome caused by marked cytokine release [33]. The severe form of the disease is characterised by haemolytic anaemia and severe acid-base derangements [34], with secondary multiple organ failure and complications such as acute renal failure, hepatopathy with marked icterus, hypoglycaemia [35], acute respiratory distress syndrome, cerebral pathology and additional immune-mediated erythrocyte destruction [32,36]. Mortality is around 12% [32]. A feature of the disease is that, in contrast to babesiosis in other domestic species, pups and immature dogs are also badly affected [32,37].

In contrast, *B. vogeli *infection in mature dogs is often unapparent or results in moderate clinical signs only, with the parasitaemia appearing to be low [32]. Although subclinical infections are common in adult dogs, pups tend to present with marked anaemia [38]. *Babesia canis *infection results in a more varied pathogenicity, intermediate between the other two species [32].

The question arises, therefore: why is *B. rossi *more virulent than the other two taxa? It is contended that, from an evolutionary perspective, domestic dogs have not had sufficient time to adapt to this parasite. Let us consider some examples where parasite and host apparently evolved together.

### Endemic stability in bovine babesiosis

*Babesia bovis *and *Babesia bigemina *are major causative agents of bovine babesiosis, especially in tropical and subtropical regions, where they are thought to have evolved [39]. Although some other hosts can be infected, usually artificially, both species are primary parasites of cattle [39]. Similarly, the vectors involved, at least in the Old World, are the bovine-specific *Rhipicephalus *(*Boophilus*) *microplus *and *Rhipicephalus *(*Boophilus*) *decoloratus *[40]. It can be concluded, therefore, that co-evolution occurred between host, vector and parasite. This is further borne out by the fact that all domestic cattle have an inherent capacity to cope with infection and to develop immunity to disease without showing clinical signs [41]. This capacity is age-specific, however, and has generally disappeared by the time a calf is one year of age. Under natural conditions, i.e. where no vector control is practised, the parasites would circulate freely in the cattle population. For the first few months of life, calves would be passively protected by maternal antibody ingested with colostrum. Once this protection has waned, calves becoming naturally infected will develop immunity without showing overt clinical signs. The net result is a high prevalence of infection, but a low occurrence of clinical disease. This situation is referred to as endemic stability. This feature is utilised to the advantage of livestock owners: calves vaccinated between 3 and 9 months of age usually develop solid immunity, without showing overt clinical signs [42].

### Babesiosis in wildlife

Although not as well studied as in cattle, there is mounting evidence that a similar situation exists in various wildlife species. For instance, black rhinoceroses (*Diceros bicornis*) have been found to harbour *Babesia bicornis *without showing any clinical signs of infection [43]. If black rhinoceroses are stressed, however, either through human intervention such as capture or under natural conditions such as drought, clinical babesiosis may ensue [43-45]. This is attributed to immunosuppression due to stress.

Similarly, a free-ranging sable antelope (*Hippotragus niger*) that had been captured in the low-lying, warm, bushveld region of north-eastern South Africa and transferred to the Johannesburg Zoo, at an altitude of 1800 m, in mid-winter succumbed to babesiosis within a few weeks [46]. It can be assumed that this individual had been a subclinical carrier of the infection. Subsequently, sable antelopes raised in captivity in Europe and therefore never exposed to infection, were translocated to a game ranch in South Africa, where they contracted babesiosis when housed in an enclosure adjoining one in which wild-caught local sable antelopes were held [47].

A further example is *Babesia leo*, which is prevalent in free-ranging lion populations [48,49]. The death of the famous lioness "Elsa" due to babesiosis after being released in the wild was probably stress-induced [50]. This lioness had repeatedly been severely mauled by the resident lion population [51].

This seems to suggest that a state of equilibrium, i.e. endemic stability, exists, at least in the three examples given. Individuals are able to cope with infection without developing overt clinical signs. This may be dependent on exposure at a young age, as suggested by the captive-bred sable antelopes being fully susceptible to infection, although stress caused by translocation may also have played a role.

How does this pertain to *Babesia *infections in canids?

### *Babesia rossi *infection in African canids

*Babesia rossi *was first described from a side-striped jackal (*Canis adustus*) in Kenya, and was subsequently also found in blood and organ smears from a jackal pup [22,52]. The literature on babesiosis in indigenous African canids is meagre. The first attempt to infect two black-backed jackals (*Canis mesomelas*) was deemed unsuccessful, as the animals remained clinically normal and subinoculation of their blood to susceptible dogs yielded no results [53]. Subsequently, black-backed jackals artificially infected by subinoculation of blood from infected domestic dogs developed parasitaemia but no overt clinical signs [54,55]. Although fatal clinical babesiosis was reported in an African wild dog (*Lycaon pictus*) pup in the Johannesburg Zoo [56], experimental subinoculation of blood from infected domestic dogs did not precipitate clinical signs in wild dogs [55]. In a large breeding centre, 17/227 (7.0%) of wild dog blood specimens were positive for *B. rossi *[57]. Clinical babesiosis had never been observed in this population, and the infected wild dogs seemed to harbour *B. rossi *with no untoward effect. Trophozoites presumed to be *Babesia canis *(probably *B. rossi*) were seen in 2/29 (6.9%) of wild dog blood smears from the Kruger National Park, South Africa [58], while a single piroplasm, possibly a *Babesia *sp., was seen on a blood smear from 1/16 wild dogs in Serengeti ecosystem, Tanzania [59]. This scant evidence fits the general picture, however, and would seem to suggest that indigenous African canids are able to cope with *B. rossi *infection and become subclinical carriers of the parasite.

*Haemaphysalis elliptica*, the only known vector of *B. rossi*, is common on South Africa domestic dogs, especially those in peri-urban or rural areas. It is rarely recovered from indigenous canids, but has been reported from captive and free-ranging wild dogs [58,60]. It is much more commonly found on indigenous felids [61-64]. It may be significant that the largest number of *H. elliptica *recovered from an indigenous canid was from a side-striped jackal, the host from which *B. rossi *was originally described [61]. Clinical babesiosis is diagnosed in around 12% of dogs presented at the Onderstepoort Veterinary Academic Hospital, where *H. elliptica *is the most common tick on dogs, while babesiosis was uncommon at another veterinary clinic, situated in a densely populated urban area around 20 km away, where *R. sanguineus *was the most common dog tick recovered [62,65].

In a recent paper on pathogens of domestic and wild canids in Brazil, antibodies to *Babesia *(no species mentioned) were found in domestic dogs only, and not in a small sample of maned wolves (*Chrysocyon brachyurus*), crab-eating foxes (*Cerdocyon thous*) and hoary foxes (*Lycalopex vetulus*) [66]. This may suggest that South American canids are not susceptible to the various *Babesia *species associated with domestic dogs.

### Dogs not indigenous to Africa

With the exception of domestic cats (descended from wild cats (*Felis silvestris*), which range throughout Africa and Eurasia) [67] and donkeys [descended from African wild asses (*Equus asinus*)] [68], all domestic animals are exotic to Africa. Domestic dogs have descended from wolves (*Canis lupus*), whose natural distribution is the Palearctic and Nearctic regions [69]. Recent mtDNA data suggest that dogs were domesticated south of the Yangtze River in China, from numerous wolves, less than 16,300 years ago [70].

There is ample evidence that natural selection is facilitating the ability of domestic animals to cope with various sub-Saharan vector-borne pathogens, provided that sufficient time has elapsed since their introduction to the continent. Although not fully resistant, West African Ndama cattle and certain goat and sheep breeds are regarded as tolerant to *Trypanosoma *infections transmitted by tsetse flies (*Glossina *species) [71]. Local breeds of *Bos indicus *(Zebu) cattle, e.g. Nguni and Sanga, exhibit a measure of inherited resistance to heartwater (*Ehrlichia ruminantium *infection, transmitted by *Amblyomma *ticks), probably acquired through millennia of natural selection [72]. This resistance does not prevent infection becoming established, but reduces the severity of clinical disease [72]. *Theileria parva*, primarily transmitted by *Rhipicephalus appendiculatus*, is a natural parasite of African buffaloes which is so virulent to cattle that the host usually succumbs before the parasite can complete its life cycle. This is the manifestation usually encountered in South Africa, where clinical manifestation is called Corridor Disease [73]. In East Africa, where natural selection has resulted in a certain degree of tolerance developing in cattle, the parasite can complete its life cycle in the host and cattle-to-cattle transmission is the norm. This manifestation is called East Coast fever [74].

Although not indigenous to Africa, genetic characterisation of the Africanis breed, which is endemic in rural areas in South Africa, supports archaeological evidence that dogs were introduced from the Middle East thousands of years ago [75]. A crucial part of the puzzle, which is still lacking, is whether natural selection in Africanis dogs has rendered them resistant or at least tolerant of *B. rossi*.

Most dogs presented to veterinary facilities in South Africa are breeds originating in Europe, Asia and the Americas [37]. As such, they are confronted by an alien parasite, *B. rossi*, and are fully susceptible.

## Conclusion

As *B. vogeli *is the least virulent of the species, I contend that it has had the longest association with domestic dogs. It is tempting to speculate that *B. vogeli*, or a closely related form, occurs in wolves, the ancestors of domestic dogs. Unfortunately, information on haemoparasites of wolves appears to be lacking. A further interesting phenomenon is that *Rhipicephalus sanguineus*, the vector of *B. vogeli*, feeds primarily on domestic dogs [76]. *R. sanguineus *has been recorded from captive wolves [77], but seemingly not from free-ranging wolves, although the literature is scanty. Could this imply that *R. sanguineus *has evolved as a dog-specific species during the thousands of years since the domestication of the dog?

It is difficult to speculate where *B. canis *fits into the picture. The widely spread golden jackal (*Canis aureus*) does occur in south-eastern Europe and could possibly be a contender for original host, but this is pure conjecture, as data on haemoparasites and ticks associated with this host are lacking.

It can be confidently stated, though, that *B. rossi *is a natural parasite of indigenous African canids. The vast majority of affected domestic dogs are fairly recent introductions to South Africa that have not yet evolved mechanisms to cope with this infection. As clinically affected dogs are usually treated and generally recover, natural selection is effectively negated and the status quo will probably be maintained indefinitely.

## Declaration of competing interests

The author declares that they have no competing interests.

## References

[B1] Collett MG Survey of canine babesiosis in South Africa J S Afr Vet Assoc 2001 71 3 180 186 10.4102/jsava.v71i3.710 11205168

[B2] Shakespeare AS The incidence of canine babesiosis amongst sick dogs presented to the Onderstepoort Veterinary Academic Hospital J S Afr Vet Assoc 1995 66 4 247 250 8691416

[B3] Hutcheon D Diseases amongst dogs. Malignant jaundice or bilious fever of the dog Agric J Cape Good Hope 1893 6 476 477

[B4] Lounsbury CH Transmission of malignant jaundice of the dog by a species of tick Agric J Cape Good Hope 1901 19 714 724

[B5] Lounsbury CH Ticks and malignant jaundice of the dog J Comp Path Therap 1904 17 113 129

[B6] Theiler A Beitrag zur Frage der Immunität bei der Piroplasmosis des Hundes Zbl Bakt Parasitenk Inf (Erste Abt/Orig) 1904 37 401 405

[B7] Gonder R Atoxylversuche bei der Piroplasmose der Hunde Arb K Gesundgheitsamte 1908 27 301 309

[B8] Nuttall GHF Hadwen S The successful drug treatment of canine piroplasmosis, together with observation upon the effect of drugs on *Piroplasma canis* Parasitology 1909 2 156 191 10.1017/S003118200000161X

[B9] Ciuca A A propos de l'immunité du chien vis-à-vis de la piroplasmose canine (Babesiose canine) Bull Soc Path Exot 1913 6 499 501

[B10] Laveran A Nattan-Larrier Piroplasmoses canines d'Europe et d'Afrique Ann Inst Pasteur 1913 27 701 717

[B11] Apanaskevich DA Horak IG Camicas JL Redescription of *Haemaphysalis *(*Rhipistoma*) *elliptica *(Koch, 1844), an old taxon of the *Haemaphysalis *(*Rhipistoma*) *leachi *group from East and southern Africa, and of *Haemaphysalis *(*Rhipistoma*) *leachi *(Audouin, 1826) (Ixodida, Ixodidae) Onderstepoort J Vet Res 2007 74 181 208 17933361 10.4102/ojvr.v74i3.122

[B12] Christophers SR Preliminary note of development of *Piroplasma canis *in the tick Br Med J 1907 76 78 10.1136/bmj.1.2402.76 20763014 PMC2356184

[B13] Brumpt E Transmission de la piroplasmose canine tunisienne par le *Rhipicephalus sanguineus* Bull Soc Path Exot 1919 12 757 764

[B14] Brumpt E Transmission de la piroplasmose canine française par le *Dermacentor reticulatus*. Embolies parasitaires dans les capillaires de l'encéphale Bull Soc Path Exot 1919 12 651 664

[B15] Nieschultz O Wawo-Roentoe FK Einige Versuche mit *Piroplasma canis *und *Rhipicephalus sanguineus* Z Infektionskr Parasit Krankh Hyg Haustiere 1931 40 60 63

[B16] Regendanz P Reichenow E Beitrag zur Uebertragungsweise von *Babesia canis *durch Zecken Zbl Bakt Parasitenk Inf (Erste Abt/Orig) 1932 124 471 478

[B17] Uilenberg G Franssen FFJ Perie M Spanjer AAM Three groups of *Babesia canis *distinguished and a proposal for nomenclature Vet Q 1989 11 33 40 2655263 10.1080/01652176.1989.9694194

[B18] Piana GP Galli-Valerio B Su di un' infezione del cane con parasiti endoglobulari Mod Zooiatr 1895 6 163 169

[B19] Matjila PT Nijhof AM Taoufik A Houwers D Teske E Penzhorn BL de Lange T Jongejan F Autochthonous canine babesiosis in The Netherlands Vet Parasit 2005 131 23 29 10.1016/j.vetpar.2005.04.020 15923085

[B20] Oines O Storli K Brun-Hansen H First case of babesiosis caused by *Babesia canis canis *in a dog from Norway Vet Parasit 2010 171 350 353 10.1016/j.vetpar.2010.03.024 20378251

[B21] Reichenow E Ueber die Entwicklung von *Theileria parva*, dem Erreger des Küstenfiebers der Rinder, in *Rhipicephalus appendiculatus* Zbl Bakt Parasitenk Inf (Erste Abt/Orig) 1937 140 223 226

[B22] Nuttall GHF On haematozoa occurring in wild animals in Africa. *Piroplasma rossi *n. sp. and *Haemogregarina canis adusti *n.sp. found in the jackal Parasitology 1910 3 108 116 10.1017/S0031182000001955

[B23] Carret C Walas F Carcy B Grande N Précigout É Moubri K Schetters TP Gorenflot A *Babesia canis canis*, *Babesia canis vogeli*, *Babesia canis rossi*: Differentiation of the three subspecies by a restricted fragment length polymorphism analysis on amplified small subunit ribosomal RNA genes J Eukaryot Microbiol 1999 46 298 303 10.1111/j.1550-7408.1999.tb05128.x 10377990

[B24] Birkenheuer AJ Neel J Ruslander DS Levy MG Breitschwerdt EB Detection and molecular characterization of a novel large *Babesia *species in a dog Vet Parasit 2004 124 151 160 10.1016/j.vetpar.2004.07.008 15381295

[B25] Matjila PT Penzhorn BL Bekker CPJ Nijhof AM Jongejan F First record of *Babesia canis vogeli *in domestic dogs in South Africa Vet Parasit 2004 122 119 125 10.1016/j.vetpar.2004.03.019 15177716

[B26] Matjila PT Leisewitz AL Jongejan F Penzhorn BL Molecular detection of tick-borne protozoal and ehrlichial infections in domestic dogs in South Africa Vet Parasit 2008 155 152 157 10.1016/j.vetpar.2008.04.012 18502588

[B27] Patton WS Preliminary report on a new piroplasm (*Piroplasma gibsoni *n. sp.) found in the blood of the hounds of the Madras hunt and subsequently discovered in the blood of the jackal, *Canis aureus* Bull Soc Path Exot 1910 3 274 280

[B28] Kjemtrup AM Wainwright K Miller M Penzhorn BL Carreno RA *Babesia conradae*, sp. nov., a small canine *Babesia *identified in California Vet Parasit 2006 138 103 111 10.1016/j.vetpar.2006.01.044 16524663

[B29] Zahler M Rinder H Schein E Gothe R Detection of a new pathogenic *Babesia microti*-like species in dogs Vet Parasit 2000 89 241 248 10.1016/S0304-4017(00)00202-8 10760414

[B30] Camacho AT Pallas E Gestal JJ Guitian FJ Olmeda AS Goethert HK Telford SR Infection of dogs in north-west Spain with a *Babesia microti*-like agent Vet Rec 2001 149 552 555 10.1136/vr.149.18.552 11720208

[B31] Matjila PT Leisewitz AL Oosthuizen MC Jongejan F Penzhorn BL Detection of a *Theileria *species in dogs in South Africa Vet Parasit 2008 157 34 40 10.1016/j.vetpar.2008.06.025 18687528

[B32] Schoeman JP Canine babesiosis Onderstepoort J Vet Res 2009 76 59 66 19967929

[B33] Taboada J Lobetti RG Greene CE Babesiosis Infectious diseases of the dog and cat 2006 3 St Louis: Saunders Elsevier 722 736

[B34] Leisewitz AL Jacobson LS de Morais HS Reyers F The mixed acid-base disturbances of severe canine babesiosis J Vet Int Med 2001 15 445 452 10.1111/j.1939-1676.2001.tb01573.x 11596731

[B35] Keller N Jacobson LS Nel M de Clerq M Thompson PN Schoeman JP Prevalence and risk factors of hypoglycemia in virulent canine babesiosis J Vet Int Med 2004 18 265 270 10.1111/j.1939-1676.2004.tb02544.x 15188810

[B36] Jacobson LS The South African form of severe and complicated canine babesiosis: clinical advances 1994-2004 Vet Parasit 2006 138 126 139 10.1016/j.vetpar.2006.01.047 16503090

[B37] van Zyl M Lewis BD, Jacobson LS An epidemiological investigation into hospitalised cases of canine babesiosis Proceedings of a symposium on canine babesiosis 1995 Onderstepoort: Faculty of Veterinary Science, University of Pretoria 42 43

[B38] Irwin PJ Hutchinson GW Clinical and pathological findings of *Babesia *infection in dogs Austr Vet J 1991 68 204 209 10.1111/j.1751-0813.1991.tb03194.x 1888313

[B39] De Vos AJ de Waal DT Jackson LA Coetzer JAW, Tustin RC Bovine babesiosis Infectious Diseases of Livestock 2004 1 2 Cape Town: Oxford University Press 406 424

[B40] Norval RAI Horak IG Coetzer JAW, Tustin RC Vectors: Ticks Infectious Diseases of Livestock 2004 1 2 Cape Town: Oxford University Press 3 42

[B41] Trueman KF Blight GW The effect of age on resistance of cattle to *Babesia bovis* Austr Vet J 1978 54 301 305 10.1111/j.1751-0813.1978.tb02465.x 687298

[B42] De Vos AJ Epidemiology and control of bovine babesiosis in South Africa J S Afr Vet Assoc 1979 50 357 362 576018

[B43] Nijhof A Penzhorn BL Lynen G Mollel JO Bekker C Jongejan F *Babesia bicornis *sp. n. and *Theileria bicornis *sp. n.: Tick-borne parasites associated with mortality in the black rhinoceros (*Diceros bicornis*) J Clin Microbiol 2003 41 2249 2254 10.1128/JCM.41.5.2249-2254.2003 12734294 PMC154668

[B44] McCullogh B Achard PL Mortalities associated with capture, translocation, trade and exhibition of black rhinoceroses Int Zoo Yb 1960 9 184 195 10.1111/j.1748-1090.1969.tb02681.x

[B45] Mugera GM Wandera JG Degenerative myopathies in East African domestic and wild animals Vet Rec 1967 80 410 413 10.1136/vr.80.13.410 6068391

[B46] Martinaglia G Red-water (babesiosis) in a sable antelope J S Afr Vet Med Assoc 1930 1 4 41 42

[B47] McInnes EF Stewart CG Penzhorn BL Meltzer DGA An outbreak of babesiosis in imported sable antelope (*Hippotragus niger*) J S Afr Vet Assoc 1991 62 30 32 2051446

[B48] Penzhorn BL Kjemtrup AE López-Rebollar LM Conrad PA *Babesia leo *n. sp. from lions in the Kruger National Park, South Africa, and its relation to other small piroplasms J Parasit 2001 87 681 685 10.2307/3285112 11426735

[B49] Bosman AM Venter EH Penzhorn BL Occurrence of *Babesia felis *and *Babesia leo *in domestic cats and various wild felid species in Southern Africa, based on reverse line blot analysis Vet Parasit 2007 144 33 38 10.1016/j.vetpar.2006.09.025 17084029

[B50] Barnett SF Brocklesby DW Some piroplasms of wild animals Symp Zool Soc Lond 1968 24 159 176

[B51] Adamson J Living free 1962 London: Collins & Harvill

[B52] Nuttall GHF Note on *Rossiella rossi *(Nuttall, 1910) occurring in the jackal in British East Africa Parasitology 1912 5 61 64 10.1017/S0031182000000093

[B53] Nuttall GHF Graham-Smith GS Note on attempts to infect the fox and the jackal with *Piroplasma canis* Parasitology 1909 2 211 214 10.1017/S0031182000001670

[B54] Neitz WO Steyn HP The transmission of *Babesia canis *(Piana and Galli-Valerio, 1895) to the black-backed jackal [*Thos mesomelas *(Schreber)], with a discussion of the classification of the piroplasms of the Canidae J S Afr Vet Med Assoc 1947 18 1 12

[B55] van Heerden J The transmission of *Babesia canis *to the wild dog (*Lycaon pictus*) (Temminck) and black-backed jackal *Canis mesomelas *(Schreber) J S Afr Vet Assoc 1980 18 119 120 7252967

[B56] Colly LP Nesbit JW Fatal acute babesiosis in a juvenile wild dog J S Afr Vet Assoc 1992 63 36 38 1569540

[B57] Matjila PT Leisewitz AL Jongejan F Bertschinger HJ Penzhorn BL Molecular detection of *Babesia rossi *and *Hepatozoon *sp. in African wild dogs (*Lycaon pictus*) in South Africa Vet Parasit 2008 157 123 127 10.1016/j.vetpar.2008.07.016 18752897

[B58] Van Heerden J Mills MGL van Vuuren MJ Kelly PJ Dreyer MJ An investigation into the health status and diseases of wild dogs (*Lycaon pictus*) in the Kruger National Park J S Afr Vet Assoc 1995 66 18 27 7629782

[B59] Peirce MA Laurenson MK Gascoyne SC Hepatozoonosis in cheetahs and wild dogs in the Serengeti ecosystem Afr J Ecol 1995 33 273 275 10.1111/j.1365-2028.1995.tb00806.x

[B60] Van Heerden J Disease and mortality of captive hunting dogs *Lycaon pictus* S Afr J Wildl Res 1986 16 7 11

[B61] Horak IG Jacot-Guillarmod A Moolman LC de Vos V Parasites of domestic and wild animals in South Africa. XXI. Ixodid ticks on domestic dogs and on wild carnivores Onderstepoort J Vet Res 1987 54 573 580 3444612

[B62] Horak IG Ixodid ticks collected at the Faculty of Veterinary Science, Onderstepoort, from dogs diagnosed with *Babesia canis *infection J S Afr Vet Assoc 1995 66 170 171 8596189

[B63] Horak IG Braack LEO Fourie LJ Walker JB Parasites of domestic and wild animals in South Africa. XXXVIII. Ixodid ticks collected from 23 wild carnivore species Onderstepoort J Vet Res 2000 67 239 250 11206391

[B64] Horak IG Heyne H Donkin EF Parasites of domestic and wild animals in South Africa. XLVIII. Ticks (Acari: Ixodidae) infesting domestic cats and wild felids in Southern Africa Onderstepoort J Vet Res 2010 77 1 Art. #3, 7 pages 10.4102/ojvr.v77i1.3 23327159

[B65] Bryson NR Horak IG Höhn EW Louw JP Ectoparasites of dogs belonging to people in resource-poor communities in north West Province, South Africa J S Afr Vet Assoc 2000 71 175 179 11205167 10.4102/jsava.v71i3.709

[B66] Curi NHDA Araújo AS Campos FS Lobato ZIP Gennari SM Marvulo MFV Silva JCR Talamoni SA Wild canids, domestic dogs and their pathogens in Southeast Brazil: disease threats for canid conservation Biodivers Conserv 2010 19 3513 3524 10.1007/s10531-010-9911-0 PMC7088301 32214695

[B67] Driscoll CA Menotti-Raymond M Roca AL Hupe K Johnson WE Geffen E Harley EH Delibes M Pontier D Kitchener AC Yamaguchi N O'Brien SJ Macdonald DW The Near Eastern origin of cat domestication Science, NY 2007 317 519 523 10.1126/science.1139518 PMC5612713 17600185

[B68] Kimura B Marshall FB Chen SY Rosenbom S Moehlman PD Tuross N Sabin RC Peters J Barich B Yohannes H Kebede F Teclai R Beja-Pereira A Mulligan CJ Ancient DNA from Nubian and Somali wild ass provides insights into donkey ancestry and domestication Proc R Soc B-Biol Sci 2011 278 50 57 10.1098/rspb.2010.0708 PMC2992715 20667880

[B69] Mech LD Canis lupus Mammalian Species 1974 37 1 6 10.2307/3503924

[B70] Pang JF Kluetsch C Zou XJ Zhang AB Luo LY Angleby H Ardalan A Ekstrom C Skollermo A Lundeberg J Matsumura S Leitner T Zhang YP Savolainen P mtDNA data indicate a single origin for dogs south of Yangtze River, less than 16,300 years ago, from numerous wolves Mol Biol Evol 2009 26 2849 2864 10.1093/molbev/msp195 19723671 PMC2775109

[B71] Connor RJ Van den Bossche P Coetzer JAW, Tustin RC African animal trypanosomoses Infectious Diseases of Livestock 2004 1 2 Cape Town: Oxford University Press 251 296

[B72] Uilenberg G Heartwater (*Cowdria ruminantium *infection): current status Adv Vet Sci Comp Med 1983 27 427 480 6359836

[B73] Lawrence JA Perry BD Williamson SM Coetzer JAW, Tustin RC Corridor disease Infectious Diseases of Livestock 2004 1 2 Cape Town: Oxford University Press 468 471

[B74] Lawrence JA Perry BD Williamson SM Coetzer JAW, Tustin RC East Coast Fever Infectious Diseases of Livestock 2004 1 2 Cape Town: Oxford University Press 448 467

[B75] Greyling LM Grobler PJ Van der Bank HF Kotze A Genetic characterisation of a domestic dog *Canis familiaris *breed endemic to South African rural areas Acta Theriol 2004 49 369 382 10.1007/BF03192535

[B76] Dantes-Torres F The brown dog tick, *Rhipicepalus sanguineus *(Latreille, 1806) (Acari: Ixodidae): From taxonomy to control Vet Parasit 2008 152 172 185 10.1016/j.vetpar.2007.12.030 18280045

[B77] Harvey JM Simpson CF Gaskin JM Sameck JH Ehrlichiosis in wolves, dogs and wolf-dog crosses J Am Vet Med Assoc 1979 175 901 905 521367

